# Curriculum Innovation: How Effective Is a Telementoring Virtual Epilepsy Curriculum in Postgraduate Medical Education? A Randomized-Controlled Educational Trial

**DOI:** 10.1212/NE9.0000000000200259

**Published:** 2025-11-14

**Authors:** Mohammed Azib AlQahtani, Annie Jiwan, Lauren Strasser, Savithiri Ratnapalan, Elizabeth J. Donner

**Affiliations:** 1 Division of Neurology, The Hospital for Sick Children, Toronto, ON, Canada;; 2 Department of Paediatrics, University of Toronto, Toronto, ON, Canada;; 3 King Fahad Specialist Hospital, Dammam, Saudi Arabia; and; 4 Learning Institute, The Hospital for Sick Children, Toronto, ON, Canada.

## Abstract

**Background and Objectives:**

Epilepsy is a complex neurologic disorder requiring specialized knowledge for effective management. Poorly controlled seizures have a significant effect on people with epilepsy and health care systems. Project Extension for Community Healthcare Outcomes (ECHOs) is a telementoring model designed to extend specialized care through virtual teaching. This study aims to evaluate the effectiveness of an interinstitutional Project ECHO-based epilepsy curriculum on the knowledge (Kirkpatrick level 2) and self-assessed effect on practice (Kirkpatrick levels 3) of neurology trainees in Canada.

**Methods:**

This randomized controlled trial included neurology trainees across Canada enrolled in Royal College of Physicians and Surgeons–accredited programs during the 2024 and 2025 academic year. Participants were recruited through program directors and resident site leads. Consenting participants were randomized using computer-generated random numbers into 2 groups. The intervention consisted of ten 1-hour weekly sessions through Zoom, including didactic presentations and case-based discussions. The curriculum was built based on the Methodology of Thomas and Kern of curriculum development. The primary outcome was improvement in objective knowledge, measured by a 30 multiple-choice quiz. Secondary outcomes included self-perceived confidence and effect on clinical practice, assessed through precourse and postcourse surveys.

**Results:**

A total of 19 participants were included in the study, with 11 in the control group and 8 in the intervention group. The intervention group achieved significantly higher quiz scores (mean score 83.3%, SD = 8) compared with the control group (mean score 64.5%, SD = 4.3), with a mean difference between groups of 18.8 percentage points (*p* = 0.004, 95% CI 6.9–30.6), corresponding to a difference of 1.3 standard deviations. Postcourse surveys indicated high satisfaction with the educational program, with 88.9% agreeing or strongly agreeing that participation was worthwhile. Significant improvements were noted in self-perceived knowledge and confidence in assessing candidacy for nonmedication treatments.

**Discussion:**

This study demonstrates the efficacy of the interinstitutional Project ECHO-based epilepsy curriculum in enhancing knowledge and self-assessed effect on practice among neurology trainees. The positive outcomes provide valuable insights into the effectiveness and feasibility of interinstitutional interactive virtual learning platforms in postgraduate medical education. Future research should explore expanding this program to other health care providers who manage epilepsy.

## Introduction and Problem Statement

Epilepsy is a prevalent complex neurologic disorder affecting 6–10 per 1,000 people worldwide.^1^ It requires specialized knowledge and skills for effective management due to the complexity and variability of the condition.^2^ Despite advancements in treatment, epilepsy remains a significant burden due to its complexity and variability.^3^ Poorly managed epilepsy carries a huge burden related to reduced quality of life, higher rate of hospitalization, and the risk of death directly related to seizures or Sudden Unexpected Death in Epilepsy.^4-9^ In addition, people with inadequately managed epilepsy face a significant social burden due to the negative social stigma associated with seizures.^2,5^ From a financial perspective, a patient with poorly managed epilepsy costs the health system an additional US$9,399 compared with a well-managed patient.^8^ The disorder's heterogeneity and the rapidly evolving treatment options necessitate a high level of expertise, which is often inconsistent among health care providers and centers. Neurology trainees, including trainees in both adult and pediatric neurology streams, are expected to be competent in diagnosing and treating epilepsy.^10^

Interinstitutional telementoring has been a rising trend over the past 5 years in the field of Neurology, which has probably been accelerated by the pandemic.^11-14^ The small literature exploring the utility of telementoring remains of small scope and intrainstitutional, posing serious questions regarding the efficacy and sustainability of larger scale and wider scope programs and curricula.^15-17^ In the field of Neurology in Canada, we have observed many initiatives under the umbrella of known national institutions such as the Canadian Association of Child Neurology (CACN), Canadian League Against Epilepsy (CLAE), and the Canadian Neurological Sciences Federation. Most of the models we have observed are hybrid, including a neuromuscular course hosted by McGill University, and a national residents' course on movement disorders delivered by the Canadian Movement Disorders Society. Other courses are provided entirely virtual such as an EEG teaching course delivered by the Canadian Epilepsy Teaching Network under the CLAE and the CACN National Academic Half Day held quarterly.

Project Extension for Community Healthcare Outcomes (ECHOs) is a telementoring model designed to extend specialized care to underserved communities through virtual teaching and collaborative learning.^18-21^ Grounded in social learning theory, the model involves interdisciplinary experts and participants engaging in didactic and case discussions, fostering an “all teach, all learn” environment.^22^ Previous small pilot studies in postgraduate medical education (PGME) have demonstrated the effectiveness of the Project ECHO model in improving the knowledge and skills in oncology, palliative care, mental health, and ophthalmology.^11,23-26^ The effect of Project ECHO-based curriculum on epilepsy management training, especially at the postgraduate medical education level, remains underexplored. In addition, there is a lack of robust national-level (or interinstitutional) evidence to demonstrate the utility and effectiveness of virtual-delivered interactive interinstitutional medical education.

In this project, we evaluate the effectiveness of Project ECHO Epilepsy: Neurology Trainee Program (NTP), a curriculum built by Project ECHO ON: Epilepsy Across Life Span for Neurology trainees across Canada. The curriculum consists of 10 sessions based on the methodology of Project ECHO, with topics and objectives determined following the Methodology of Thomas and Kern and based on a needs assessment survey and the Royal College of Physicians and Surgeons of Canada (RCPSC) stated objectives of Neurology training.^10,27^

### Hypothesis and Objectives

The aim of this project is to assess the effect of a Project ECHO-based epilepsy curriculum on the knowledge (Kirkpatrick level 2) and self-assessed effect on practice (Kirkpatrick level 3) of neurology trainees in Canada in managing epilepsy.^28^ We hypothesize that participation in the Project ECHO-based epilepsy curriculum will increase the average quiz scores of participants by at least 10%.

## Methods and Curriculum Description

### Curriculum Development

The curriculum was developed using the Six-Step Approach of Thomas and Kern to Curriculum Development.^27^ We used a mixed methodology model to identify the needs of Neurology trainees across Canada, following these steps.

### General Needs Assessment

A qualitative method was used to explore the problem and establish a starting point. Unstructured individual interviews were conducted with a convenience sample of epilepsy experts, including 2 physicians, an epilepsy fellow, a nurse practitioner, and a registered nurse. The primary question asked was: “What do you think a neurology trainee needs to know about epilepsy management?”

The responses from the interviews were collected and entered by one of the authors into an electronic document. Thematic analysis was used to identify common themes and patterns within the responses. Key phrases and concepts were identified and coded. Each code represented a specific competency or topic mentioned by the participants. The codes were grouped into broader themes that encapsulated the main areas of knowledge and skills required for epilepsy management and compiled into a comprehensive list of competencies and topics, which served as the foundation for the targeted needs assessment. This detailed analysis ensured that the curriculum development was grounded in the actual needs identified by practitioners involved in the everyday treatment of patients with epilepsy, providing a robust basis for the subsequent steps in the project.

### Targeted Needs Assessment

A survey was developed in collaboration with the Project ECHO Epilepsy Hub team and supported by an interdisciplinary education specialist (Appendix A). This survey was based on the general needs assessment. It was distributed to all centers with a neurology residency program across Canada through program directors and resident site leads—a group of 10 residents across Canada who volunteered to represent their centers. All trainees in eligible adult and pediatric neurology programs received the survey link. A total of 30 responses were received. A focused group consisting of a convenience sample of 10 residents across Canada (the resident site leads) and an interdisciplinary education specialist reviewed the survey results. Together, this group developed the suggested topics, objectives, and recommended presenters.

### Goals and Objectives

The results of the needs assessment were compared with RCPSC competencies and then reviewed by the Project ECHO Epilepsy Educational Committee. The final version was approved by the Project ECHO Epilepsy leadership.

### Educational Strategies

An educational strategy was developed grounded in the Project ECHO framework and informed by the needs assessment.

### Implementation

Ten 1-hour weekly sessions were conducted through Zoom, facilitated by the Project ECHO Epilepsy infrastructure at The Hospital for Sick Children. The list of topics, dates, responsible teachers, and objectives is attached in Appendix B. Each session consisted of 2 parts. The first section consisted of a didactic presentation delivered over 15–20 minutes at the beginning of the session, by an invited epilepsy expert. The second part was a case-based discussion, where a trainee presented a relevant case, followed by a 30-minute discussion starting with the trainees, guided and followed by a multidisciplinary panel of epilepsy experts (i.e., Hub team members).

### Curriculum Evaluation

To evaluate the efficacy of the curriculum, we conducted a randomized controlled trial (RCT) to evaluate the effect of the Epilepsy training program in improving neurology residents' knowledge of epilepsy. The NTP program was the intervention of interest. The study included all neurology trainees across Canada enrolled in an RCPSC-accredited Adult and Pediatric Neurology program during the academic year 2024 and 2025 and who provided informed consent to participate in the study and the course. We approached all trainees through their program directors and resident site leads in the NTP planning team. Any resident involved in creating the teaching module or piloting the examination question was excluded from the study.

The primary outcome in our evaluation is improvement in objective knowledge, which correlates with level 2 in the model of Kirkpatrick for medical education.^28^ The evaluation method consisted of a 30 multiple-choice quiz developed by the research team, focusing on key aspects of epilepsy management and accounting for the objectives identified in the needs assessment. The test was piloted with 5 neurology residents and refined as needed. Blindness of the participants was maintained by limiting access to assignment groups to team members not involved in the delivery of the NTP program.

The secondary outcome, to assess self-perceived confidence and effect on clinical practice, correlating with level 3 in the Kirkpatrick model, was assessed using 2 surveys developed by the research team. A precourse and a postcourse survey was distributed to all participants.

Assuming a mean score difference of 10% between the 2 groups, the sample size needed to achieve a power of 0.8 and α 0.05; 2-tailed is 9 students per group. Volunteers were randomized using a RedCAP-generated randomization list and placed into either the pretest group (control group) or the posttest (intervention group). Although participants were aware of their group allocation, the educators and members of the study team involved in curriculum delivery were blinded to group assignments.

#### Statistical Methods

Descriptive statistics were used to summarize participant demographics, including the level of postgraduate education. Independent student *t* tests were used to compare the difference between the scores of the control and intervention groups. Descriptive statistics, independent *t* tests, and chi-square tests were used to compare the survey results as appropriate. Participants with incomplete assessments were excluded from the final analysis.

### Standard Protocol Approvals, Registrations, and Participant Consents

University of Toronto Health Sciences research ethics board approval was obtained (protocol 47679). Informed written consent was obtained from all participants in the evaluation. Confidentiality and anonymity of participants were maintained.

### Data Availability

Deidentified data and study protocol are available on request of any qualified investigator for purposes of replicating procedures and results up to 5 years from the publication of the study.

## Results and Assessment Data

**Figure 1:**
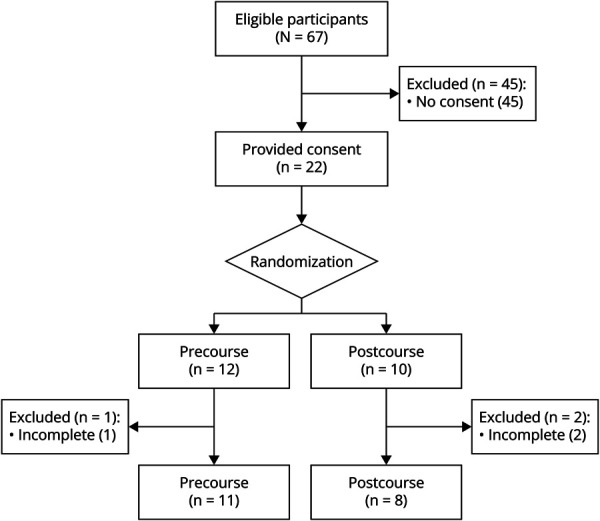
Flowchart of Participant Recruitment and Allocation This flowchart illustrates the recruitment, consent, randomization, and final inclusion of participants in the study. Of the 67 neurology trainees who registered for the program, 22 provided written informed consent and were randomized into either the intervention group (postcourse quiz) or the control group (precourse quiz). Three participants withdrew because of time constraints, resulting in a final cohort of 19 participants: 11 in the control group and 8 in the intervention group. The flowchart outlines the steps from initial registration through to final analysis.

A total of 67 participants registered in the program out of an estimated 261 total Adult and Pediatric Neurology trainees representing 25.7% of all trainees across Canada. A total of 22 participants signed the written consent and were included in the study and randomized to the intervention group and control group. Three participants dropped out because of lack of time to answer the quiz or fill out the survey. The final cohort consisted of 11 participants in the control group and 8 in the intervention group (Figure 1). The study participants represent 28% of trainees participating in the curriculum and 7.3% of all trainees across Canada.

### Demographics

The cohort consisted of trainees from adult (n = 8) and pediatric (n = 11) neurology programs. Trainees in adult neurology programs were 5 (45.5%) in the control group and 3 (37.5%) in the intervention group (*p* = 0.729). They were distributed between postgraduate years 1–5 with no difference between control and intervention groups (*p* = 0.628). Participants were enrolled in training programs in Ontario (n = 14), British Columbia (n = 2), Alberta (n = 1), Manitoba (n = 1), and Newfoundland (n = 1) (*p* = 0.377). (Table and eFigure 1).

**Table:** Demographic Characteristics of the Participants

Group	Precourse (control) group	Postcourse (intervention) group
Program	Adult stream: 5 (45.5%)	Adult stream: 3 (37.5%)
	Pediatric stream: 6 (54.5%)	Pediatric stream: 5 (62.5%)
Year of training		
PGY1	1	0
PGY2	3	3
PGY3	2	0
PGY4	2	2
PGY5	3	3
Province		
ON	10	4
NL	0	1
BC	1	1
AB	0	1
MB	0	1

Abbreviation: PGY = Postgraduate year.

This table summarizes the demographic distribution of neurology trainees who participated in the study. Participants are categorized into precourse (control) and postcourse (intervention) groups. Data are presented by training stream (adult vs pediatric), postgraduate year (PGY1–PGY5), and province of residency training. Percentages are calculated within each group.

When asked why they participated in the course, the reasons were (1) to learn best-practice care in epilepsy (100%), (2) to connect with my peers (47.4%), (3) because I like the program format (57.8%), (4) to prepare for board examinations (63.1%), and (5) I plan to pursue further training in epilepsy (e.g., epilepsy fellowship) (47.4%).

### Primary Assessment: Kirkpatrick Level 2 Evaluation

**Figure 2:**
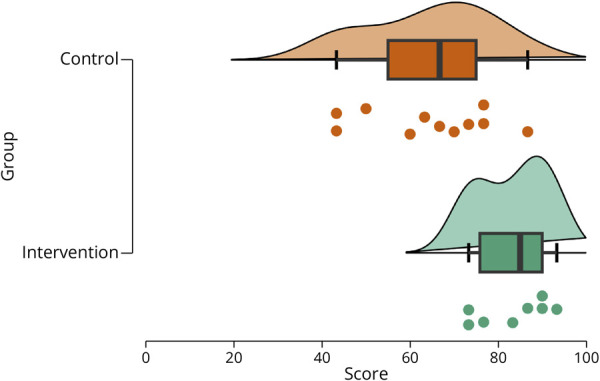
Participant Score Distribution Raincloud plots displaying the score distribution of participants in both control and intervention groups with a combined violin plot, box plot, and scatter plot providing a comprehensive view of the data distribution are used. The figure shows more homogeneity in the scores of the intervention group and demonstrates that all scores in the intervention group are higher than the median score in the control group. In addition, the new figure illustrates that the 25th quartile in the intervention group correlates with the 75th quartile in the control group.

The control group had a mean score of 64.5% (SD = 14.3). The minimum score was 43.3%, while the maximum score was 86.7%. In the intervention group, the mean score was 83.3% (SD = 8) with a minimum of 73.3% and a maximum of 93.3%. The mean difference between groups is 18.8 percentage points (*p* = 0.004, 95% CI 6.9–30.6), which is an increase of 1.3 standard deviations in the intervention group compared with the control group (Figure 2). The Shapiro-Wilk test of normality has failed to show any deviation of normality in control (*p* = 0.55) and intervention groups (*p* = 0.24). There was no difference in variance between the groups in the Levene test of equality of variances (*p* = 0.127).

Review of individual questions demonstrated no statistically significant difference between the groups in correct answers, except for the question: *Which of the following is considered a phase 2 surgical evaluation?* Where 87.5% of the intervention group compared with 36.4% in the control group answered correctly (*p* = 0.026). Each question was answered correctly by at least 4 individuals in each group.

### Secondary Assessment

All participants answered the precourse survey, while the postcourse survey was answered by 9 participants. In the postcourse survey, 88.9% agree or strongly agree with the statement: *Participating in the program has been a worthwhile experience*. Our cohort felt that both didactic lectures (33.3% agree, 55.6% strongly agree) and case discussions (22.2% agree, 66.7% strongly agree) were valuable in their learning. 88.9% of the participants were satisfied with the technology used in the ECHO sessions. When asked if they would recommend the program to their colleagues, 77.8% strongly agree and 11.1% agree.

One participant indicated that they strongly disagree with all the statements in the survey. They indicated the main barrier to their learning was the timing of the course, which was delivered at 5:30 pm EST. They did not identify what province they were joining from.

### Kirkpatrick Levels Self-Perceived Level 2 and 3 Evaluation

When the cohort was asked about their knowledge and level of confidence in ordering initial investigations, prescribing ASMs, discussing their side effects, combining them as needed, and counselling patients, all differences between precourse and postcourse groups were 10% or less with 65%–80% either agreeing or strongly agreeing they are knowledgeable or confident to do it in their practice. By contrast, when discussing their comfort with assessing candidacy for nonmedication treatments such as surgery, neurostimulation, and ketogenic diet therapy, there were significant differences between groups. In the precourse group, 21.1% disagree with the statement I know enough about this, and 36.8% are neutral, while 26.3% and 15.8% agree or strongly agree, respectively. The same group also disagree (26.3%) or is neutral (42.1%) to the statements *I'm confident enough to do this*. In the other hand, the postcourse group, 44.4% agree and 11.1% strongly agree with both statements, while the rest feel neutral. When asked if it *Improved the quality and safety of patient care for epilepsy in my practice*, 88.9% answered agree (22.2%) or strongly agree (66.7%).

## Discussion and Lessons Learned

This study evaluates the efficacy of a nationwide interinstitutional telementoring model in PGME through a RCT. This contributes much-needed evidence supporting the use of virtual platforms to deliver regional or national curricula in PGME.

From the trainees' perspective, we believe that remote interinstitutional education offers several advantages. It provides exposure to cutting-edge developments beyond their home institutions, enhancing both clinical competence and career planning. It also reduces barriers such as travel costs, time commitments, and the need to use vacation time, thereby improving accessibility, particularly for trainees with financial constraints or family responsibilities. Notably, 47% of participants valued the opportunity to connect with peers and mentors from other institutions, highlighting the importance of professional networking and supporting our view.

From the educators' standpoint, we believe that remote education can increase the return on investment of time and human resources. Smaller programs, often with fewer than 10–15 trainees, may struggle to justify the cost of organizing educational activities that are poorly attended due to clinical duties or postcall fatigue. Interinstitutional remote learning allows for broader participation without compromising group cohesion, unlike large-scale in-house sessions that combine multiple specialties. This model may also reduce the need for repeated didactic sessions, freeing educators to focus on bedside and clinical teaching. Although these benefits are recognized anecdotally, this study addresses the gap in empirical evidence regarding the feasibility and effectiveness of such models.

A key challenge in implementing interinstitutional curricula is the need for technologic infrastructure, human resources, and funding. Project ECHO, with more than 2,800 programs in more than 200 countries and regions, offers a scalable solution.^29^ Its success is underpinned by a robust evidence base, including more than 700 scholarly publications.^20,22,29,30^ The ECHO model's interactive, case-based, flipped-classroom approach—where “all teach, all learn”—addresses a major barrier to digital education: lack of interactivity.^22,26^ This aligns with findings by Petchame et al. and was echoed by our participants.^14^

Traditionally, interinstitutional medical education has relied on models such as temporary trainee relocation to participate in national in-person programs. Although these initiatives have played a valuable role in advancing neurology education, they are often constrained by logistical challenges, including venue coordination, limited capacity, and conflicting faculty schedules, as well as financial and time-related burdens. Trainees frequently must use vacation time, weekends, or designated educational days to attend. By contrast, the Project ECHO model offers a more scalable and resource-efficient alternative, enabling broader participation without the need for travel. Nonetheless, it is not without limitations, the most notable being the challenge of scheduling across multiple time zones. These 2 models are not mutually exclusive; rather, they represent complementary approaches to addressing shared educational objectives. A direct comparative evaluation of traditional and ECHO-based modalities would provide valuable insights into their relative effectiveness.

Of interest, although objective knowledge improved, postcourse surveys did not show a significant increase in confidence. This may reflect the distinction between theoretical knowledge and practical competence, as famously articulated by Sir William Osler: “To study the phenomena of disease without books is to sail an uncharted sea, while to study books without patients is not to go to sea at all.”^31^ This narrative is supported by the literature emphasizing the importance of experiential learning and clinical application. However, a notable exception was increased confidence in assessing candidacy for nonpharmacologic treatments—a topic frequently discussed in the case-based sessions.^32-35^

The timing of the course emerged as a significant barrier for trainees across different provinces as discussed by 1 survey participant. The 5:30 pm EST schedule was inconvenient for trainees across time zones—midday for those in western Canada and late evening for those in the east. This challenge, raised by both survey respondents and site leads, may hinder participation and engagement. Although asynchronous modules or time zone-specific sessions could improve accessibility, they may compromise the interactivity and national reach of the current model. This remains a complex issue without a simple solution.

Our study has several limitations. First, participants were self-selected volunteers, likely more motivated and interested in epilepsy, introducing potential selection bias and limiting generalizability. Second, we did not include a control group receiving traditional in-person education, making it difficult to isolate the effects of the ECHO model. Future research should incorporate comparative designs and more diverse cohorts to better assess the relative effectiveness of telementoring vs conventional methods.

In conclusion, our study demonstrates that a Project ECHO-based epilepsy curriculum can effectively enhance objective knowledge and perceived clinical effect among neurology trainees in Canada. Despite limitations such as volunteer bias and the absence of a traditional control group, our findings support the potential of interinstitutional virtual learning platforms in PGME. Future studies should aim for broader inclusion and direct comparisons with conventional teaching to further validate these findings. Overall, our work contributes to the growing body of evidence supporting innovative telementoring models in neurology education and beyond.

## Glossary

CACN: Canadian Association of Child Neurology
CLAE: Canadian League Against Epilepsy
ECHO: Extension for Community Healthcare Outcomes
NTP: Neurology Trainee Program
PGME: postgraduate medical education
RCPSC: Royal College of Physicians and Surgeon
RCT: randomized controlled trial

## References

[1] Fiest KM, Sauro KM, Wiebe S, et al. Prevalence and incidence of epilepsy: a systematic review and meta-analysis of international studies. Neurology. 2017;88(3):296-303. doi:10.1212/WNL.0000000000003509 27986877 PMC5272794

[2] Tellez‐Zenteno JF, Patten SB, Jetté N, Williams J, Wiebe S. Psychiatric comorbidity in epilepsy: a population‐based analysis. Epilepsia. 2007;48(12):2336-2344. doi:10.1111/j.1528-1167.2007.01222.x 17662062

[3] Chen Z, Brodie MJ, Liew D, Kwan P. Treatment outcomes in patients with newly diagnosed epilepsy treated with established and new antiepileptic drugs: a 30-Year longitudinal cohort study. JAMA Neurol. 2018;75(3):279-286. doi:10.1001/jamaneurol.2017.3949 29279892 PMC5885858

[4] Bishop M, Allen CA. The impact of epilepsy on quality of life: a qualitative analysis. Epilepsy Behav. 2003;4(3):226-233. doi:10.1016/S1525-5050(03)00111-2 12791323

[5] Strzelczyk A, Aledo-Serrano A, Coppola A, et al. The impact of epilepsy on quality of life: findings from a European survey. Epilepsy Behav. 2023;142:109179. doi:10.1016/j.yebeh.2023.109179 37058861

[6] Keller AE, Whitney R, Li SA, Pollanen MS, Donner EJ. Incidence of sudden unexpected death in epilepsy in children is similar to adults. Neurology. 2018;91(2):e107-e111. doi:10.1212/WNL.0000000000005762 29884734

[7] Singh K, Katz ES, Zarowski M, et al. Cardiopulmonary complications during pediatric seizures: a prelude to understanding SUDEP. Epilepsia. 2013;54(6):1083-1091. doi:10.1111/epi.12153 23731396 PMC5304951

[8] Cramer JA, Wang ZJ, Chang E, et al. Healthcare utilization and costs in adults with stable and uncontrolled epilepsy. Epilepsy Behav. 2014;31:356-362. doi:10.1016/j.yebeh.2013.09.046 24239435

[9] Strafford H, Hollinghurst J, Lacey AS, et al. Health care utilization and mortality for people with epilepsy during COVID-19: a population study. Epilepsia. 2024;65(5):1394-1405. doi:10.1111/epi.17920 38441332

[10] Neurology Competencies. Version 1.0. Effective for Residents Who Enter Training on or After July 1, 2020. Accessed September 12, 2024. news.royalcollege.ca/content/dam/documents/ibd/neurology/neurology-competencies-e.pdf

[11] Choi Y, Chodoff AC, Brown K, et al. Preparing future medicine physicians to care for cancer survivors: project ECHO® in a novel internal medicine and family medicine residency curriculum. J Cancer Educ. 2023;38(2):608-617. doi:10.1007/s13187-022-02161-z 35366218 PMC8976217

[12] Pfennig M, Lee A, Mi M. How does telementoring impact medical education within the surgical field? A scoping review. Am J Surg. 2022;224(3):869-880. doi:10.1016/j.amjsurg.2022.04.038 35545476 PMC9417933

[13] Bork-Hüffer T, Kulcar V, Brielmair F, et al. University students' perception, evaluation, and spaces of distance learning during the COVID-19 pandemic in Austria: what can we learn for post-pandemic educational futures? Sustainability. 2021;13(14):7595. doi:10.3390/su13147595

[14] Petchamé J, Iriondo I, Korres O, Paños-Castro J. Digital transformation in higher education: a qualitative evaluative study of a hybrid virtual format using a smart classroom system. Heliyon. 2023;9(6):e16675. doi:10.1016/j.heliyon.2023.e16675 37303520 PMC10248111

[15] Morales MY, Rodríguez GV, Pérez DG, et al. Teletraining and implementation of teleinterconsultation in oral pathology. Health service Talcahuano—Chile. Oral Surg Oral Med Oral Pathol Oral Radiol. 2020;129(1):e146. doi:10.1016/j.oooo.2019.06.630

[16] Jin ML, Brown MM, Patwa D, Nirmalan A, Edwards PA. Telemedicine, telementoring, and telesurgery for surgical practices. Curr Probl Surg. 2021;58(12):100986. doi:10.1016/j.cpsurg.2021.100986 34895561

[17] Hecker A, Nischwitz SP, Petritsch J, et al. Undergraduate skills training in pandemic times: where is the future of medical education? Eur J Investig Health Psychol Educ. 2023;13(7):1219-1228. doi:10.3390/ejihpe13070090 PMC10377890 37504481

[18] Arora S, Kalishman S, Thornton K, et al. Project ECHO (project extension for community healthcare outcomes): a national and global model for continuing professional development. J Contin Educ Health Prof. 2016;36(suppl 1):S48-S49. doi:10.1097/CEH.0000000000000097 27584072

[19] The ECHO Model. Project ECHO. Accessed October 7, 2024. projectecho.unm.edu/model/

[20] Katzman JG, Galloway K, Olivas C, et al. Expanding health care access through education: dissemination and implementation of the ECHO model. Mil Med. 2016;181(3):227-235. doi:10.7205/MILMED-D-15-00044 26926747

[21] Sockalingam S, Arena A, Serhal E, Mohri L, Alloo J, Crawford A. Building provincial mental health capacity in primary care: an evaluation of a project ECHO mental health program. Acad Psychiatry. 2018;42(4):451-457. doi:10.1007/s40596-017-0735-z 28593537

[22] Burman ME, McGee N, Proctor J, Hart AM, Moody EJ, Hardesty C. ECHO: a model for professional development in nursing through learning networks. J Contin Educ Nurs. 2021;52(4):198-204. doi:10.3928/00220124-20210315-09 34038238

[23] Bennett KA, Ong T, Verrall AM, Vitiello MV, Marcum ZA, Phelan EA. Project ECHO-Geriatrics: training future primary care providers to meet the needs of older adults. J Grad Med Educ. 2018;10(3):311-315. doi:10.4300/jgme-d-17-01022.1 29946389 PMC6008038

[24] Roy A, Das B, Garg P, Das B, Garg P, Garg P. Project ECHO (extension for community health care outcomes), an online tool for residents' education: a pilot study. Indian J Ophthalmol. 2020;68(10):2318-2319. doi:10.4103/ijo.ijo_588_20 32971712 PMC7727996

[25] Joshi A, Pathare A, Hameed U, et al. Using the project ECHO model to facilitate mental health training in graduate and undergraduate medical education: results from two pilot programs. Acad Psychiatry 2022;47(4):416-421. doi:10.1007/s40596-022-01715-z 36258083

[26] Doherty M, Abdullah QK. Using project ECHO to deliver a tele-mentoring and teaching program on palliative care in South Asia: interpretive description of participants' experiences with a community of practice for learning. Palliat Support Care. 2024;22(6):1957-1965. doi:10.1017/S1478951524000762 38736371

[27] Thomas PA, Kern DE, Hughes MT, Tackett SA, Chen BY. Curriculum Development for Medical Education: A Six-step Approach. JHU Press; 2022. 10.1097/ACM.0000000000002580 30681454

[28] Bates R. A critical analysis of evaluation practice: the Kirkpatrick model and the principle of beneficence. Eval Program Plann. 2004;27(3):341-347. doi:10.1016/j.evalprogplan.2004.04.011

[29] Project ECHO. Annual Report; 2024. Accessed May 29, 2025. hsc.unm.edu/echo/

[30] Zhou C, Crawford A, Serhal E, Kurdyak P, Sockalingam S. The impact of project ECHO on participant and patient outcomes: a systematic review. Acad Med. 2016;91(10):1439-1461. doi:10.1097/ACM.0000000000001328 27489018

[31] Amedee RG, Seoane L. From the editor's desk: sailing osler's uncharted sea with innovation and collaboration at the helm. Ochsner J. 2016;16(1):1-2. PMC4795488 27028013

[32] Ottenhoff-de Jonge MW, van der Hoeven I, Gesundheit N, van der Rijst RM, Kramer AWM. Medical educators' beliefs about teaching, learning, and knowledge: development of a new framework. BMC Med Educ. 2021;21(1):176. doi:10.1186/s12909-021-02587-x 33745444 PMC7981947

[33] Witheridge A, Ferns G, Scott-Smith W. Revisiting Miller's pyramid in medical education: the gap between traditional assessment and diagnostic reasoning. Int J Med Educ. 2019;10:191-192. doi:10.5116/ijme.5d9b.0c37 31655795 PMC7246123

[34] Dowson J. Transferring knowledge into practice? Exploring the feasibility of action learning for improving knowledge, skills and confidence in clinical communication skills. BMC Med Educ. 2019;19(1):37. doi:10.1186/s12909-019-1467-4 30691472 PMC6350350

[35] Sutoi D, Popa D, Cindrea CA, et al. The impact of a one-day multidisciplinary workshop on medical students' self-assessed confidence, knowledge, and teamwork skills: a pre-post study. Adv Med Educ Pract. 2025;16:401-410. doi:10.2147/AMEP.S509297 40166603 PMC11955773

